# Prediction of Static Characteristic Parameters of an Insulated Gate Bipolar Transistor Using Artificial Neural Network

**DOI:** 10.3390/mi13010004

**Published:** 2021-12-21

**Authors:** Qing Yao, Yufeng Guo, Bo Zhang, Jing Chen, Jun Zhang, Maolin Zhang, Xiaobo Guo, Jiafei Yao, Weihua Tang, Jianhua Liu

**Affiliations:** 1 College of Integrated Circuit Science and Engineering, Nanjing University of Posts and Telecommunications, Nanjing 210023, China; yqdeemail@163.com (Q.Y.); cjjcnjupt@163.com (J.C.); bravaisxx@163.com (J.Z.); zhangml5277@163.com (M.Z.); guoxb26@126.com (X.G.); jfyao@njupt.edu.cn (J.Y.); whtang@njupt.edu.cn (W.T.); jhliu_njupt@163.com (J.L.); 2 National and Local Joint Engineering Laboratory for RF Integration and Micro-Packaging Technologies, Nanjing University of Posts and Telecommunications, Nanjing 210023, China; 3 State Key Laboratory of Electronic Thin Films and Integrated Devices, University of Electronic Science and Technology of China, Chengdu 610054, China

**Keywords:** artificial neural network, insulated gate bipolar transistor (IGBT), breakdown voltage, on-state voltage, static latch-up immunity, threshold voltage

## Abstract

Breakdown voltage (*BV*), on-state voltage (*V*_on_), static latch-up voltage (*V*_lu_), static latch-up current density (*J*_lu_), and threshold voltage (*V*_th_), etc., are critical static characteristic parameters of an IGBT for researchers. *V*_on_ and *V*_th_ can characterize the conduction capability of the device, while *BV*, *V*_lu_, and *J*_lu_ can help designers analyze the safe operating area (SOA) of the device and its reliability. In this paper, we propose a multi-layer artificial neural network (ANN) framework to predict these characteristic parameters. The proposed scheme can accurately fit the relationship between structural parameters and static characteristic parameters. Given the structural parameters of the device, characteristic parameters can be generated accurately and efficiently. Compared with technology computer-aided design (TCAD) simulation, the average errors of our scheme for each characteristic parameter are within 8%, especially for *BV* and *V*_th_, while the errors are controlled within 1%, and the evaluation speed is improved more than 10^7^ times. In addition, since the prediction process is mathematically a matrix operation process, there is no convergence problem, which there is in a TCAD simulation.

## 1. Introduction

The insulated gate bipolar transistor (IGBT) is widely used in power electronics due to its superior performance [1,2]. Analysis of its static characteristic parameters has always been a key part of the design process, given that these parameters, such as on-state voltage (*V*_on_) and threshold voltage (*V*_th_), can characterize the conduction capability of the device [3,4]. In addition, breakdown voltage (*BV*), static latch-up voltage (*V*_lu_), and static latch-up current density (*J*_lu_) are helpful when evaluating the safe operating area of the device and its reliability [5,6,7]. Conventionally, technology computer-aided design (TCAD) simulation tools are used to obtain these characteristic parameters of the device before experimental testing because of the advantage of low prediction errors [1,2,3,4]. However, this method may suffer from non-convergence when solving the semiconductor physical equations, thus decreasing the efficiency of obtaining the characteristic parameters.

Recently, machine learning techniques for predicting the electrical characteristic parameters of the semiconductor device have been booming due to their ability to learn the relationship between structural parameters and characteristics efficiently [8,9,10,11,12,13]. However, most work is limited to providing only one characteristic parameter, such as the threshold voltage of a junctionless nanowire transistor [11] or the breakdown voltage of a lateral power device [12]. As for those that can output multiple characteristics, the characteristic parameters are extracted from the current–voltage (*I*-*V*) curve and capacitance–voltage (*C*-*V*) curve, which still relies on human-intensive operation [13]. In addition, machine learning is applied to predict the current value of IGBTs in circuits and suppress the current imbalance of parallel-connected IGBTs through artificial neural networks, or to predict the remaining lifetime of the IGBTs operating in circuits [14,15,16]. However, the focus of these works is on monitoring and optimizing IGBT operations in the circuit, rather than on exploring the relationship between the device’s structural parameters and characteristic parameters.

In this paper, we propose a simple multi-layer artificial neural network (ANN) framework to predict the vital static characteristic parameters of the IGBT, including *BV*, *V*_on_, *V*_th_, *V*_lu_, and *J*_lu_. The proposed method enables designers to predict the static characteristic parameters effectively, speeding up the device design process. After testing, for the same testing samples, the prediction speed of the ANN is improved by more than 10^7^ times compared to TCAD simulation. This method avoids the non-convergence problem of TCAD simulations, and, if supported by experimental data, the method’s predictions will be closer to the experimental test values.

## 2. Dataset Generation and Division

**Figure 1:**
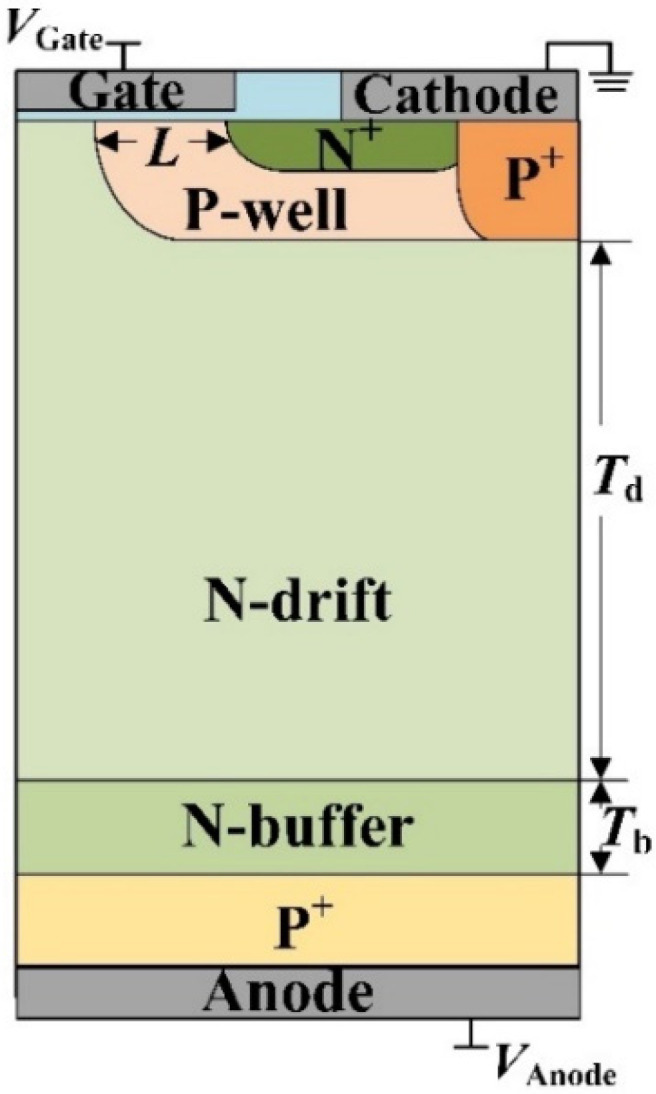
Schematic diagram of the IGBT structure.

**Figure 2:**
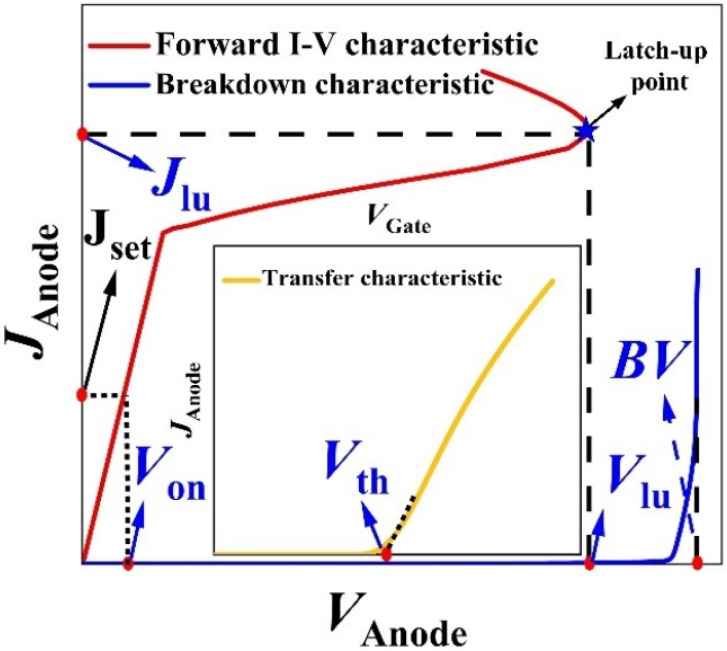
Schematic diagram of the IGBT static characteristic curves.

Figure 1 shows the structural schematic diagram of the IGBT, and Figure 2 gives the curves of its static characteristic, including the breakdown characteristic curve, forward I-V characteristic curve, and transfer characteristic curve.

As shown in the figure, the prediction targets, including *BV*, *V*_th_, *V*_on_, *V*_lu_, and *J*_lu_, are marked in the corresponding characteristic curves. In order to obtain the dataset of static characteristic parameters for IGBT with different structures, some vital structural parameters were generated randomly within a certain range. The detailed information on these structural parameters and their ranges is shown in Table 1. Moreover, during the data collection process, the static latch-up point was extracted from the forward I-V characteristic curve [7]. In addition, *V*_on_ was defined as the voltage at the anode when the anode’s current density reached a certain set value (J_set_), meaning that it could also be extracted from the forward I-V curve at a gate voltage V_G_ of 15 V, with J_set_ defined as 100 A/cm^2^ [1]. We simulated the device characteristics of multiple IGBTs with the carrier lifetime of 1µs using the TCAD tool as the total dataset [17]. After finishing the total dataset collection from the simulation, the dataset was further divided into the training set and the testing set for the training process and the testing process of the ANN framework.

## 3. Methodology

**Figure 3:**
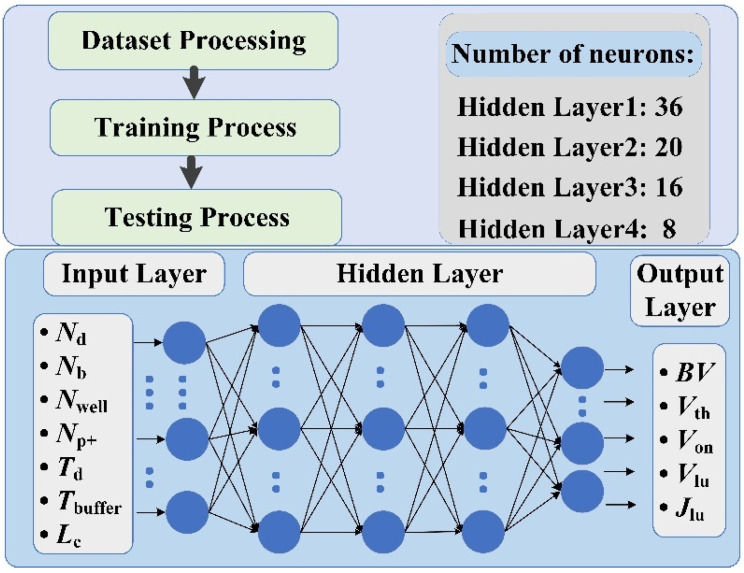
The overall flow and structure of the proposed ANN framework.

Figure 3 shows the overall flow of the proposed approach and the designed ANN framework structure. In general, the proposed scheme can be divided into three steps: dataset processing, the training process, and the testing process. It is worth noting that, since the different input parameters have different scales, this will result in the updating of the ANN weights and biases during training being more susceptible to large-scale input parameters and the effect of input parameters with small scales on the output may be ignored. We use a normalization method to compress the structural parameters to (0, 1), and the process can eliminate the effect of unit and scale differences between input parameters in order to treat each class of input parameters equally, thereby increasing the prediction accuracy and efficiency of the ANN. Similar approaches to data pre-processing have been reported in papers related to machine learning [12,13].

The ANN is a nonlinear dynamic learning system, implemented using a mathematical method, which can handle complex nonlinear prediction problems by simulating biological neural networks. It consists of an input layer, hidden layers, and an output layer. The ANN structure for the proposed method is built using the Tensorflow library [18]. To obtain a robust framework for IGBT static characteristic parameter prediction, several trials and adjustments have been conducted. Finally, the hidden layers are set to four layers, and the number of neurons in each layer is 36, 20, 16, and eight, from first to last. Such an ANN structure has sufficient fitting ability for the static performance prediction task. Meanwhile, it also can avoid overfitting problems due to its extensive learning ability.

The ANN algorithm is capable of continuously optimizing the weights and biases between each layer to minimize the error between the predicted value and the actual value during the training process. We defined the error function by using the mean absolute error (MAE) loss function [11]. Moreover, we used the Adam optimizer to update the network parameters to reduce the error [19]. Mathematically, the MAE loss function is expressed as follows:

(1) $$\mathrm{MAE}=\frac{1}{m}\sum_{i=1}^{m}\left|\left(y_{i}-{\hat{y}}_{i}\right)\right|$$

where *m* is the number of training samples, *y_i_* and *ŷ_i_* are the actual value and predicted value of the output feature of the ith training sample, respectively. Moreover, to capture the nonlinear relationship between the device structure and the static characteristic parameters, each neuron is equipped with a ReLU activation function [20].

## 4. Results Analysis

The following numerical results validate the proposed scheme. The prediction results of the ANN for different static characteristic parameters are first shown, then the effects of the different regression algorithms and the number of training samples on the prediction errors are investigated separately, and the reasons for the high and low average errors for different characteristics parameters are explained through physical theory. In addition, the ANN method has also shown its great ability to fit the trends of the static characteristic parameters well as they change with the device’s structural parameters. Finally, we reveal the advantages in prediction speed by comparing the time of using ANN and TCAD simulation.

### 4.1. Prediction Results for Characteristic Parameters

**Figure 4:**
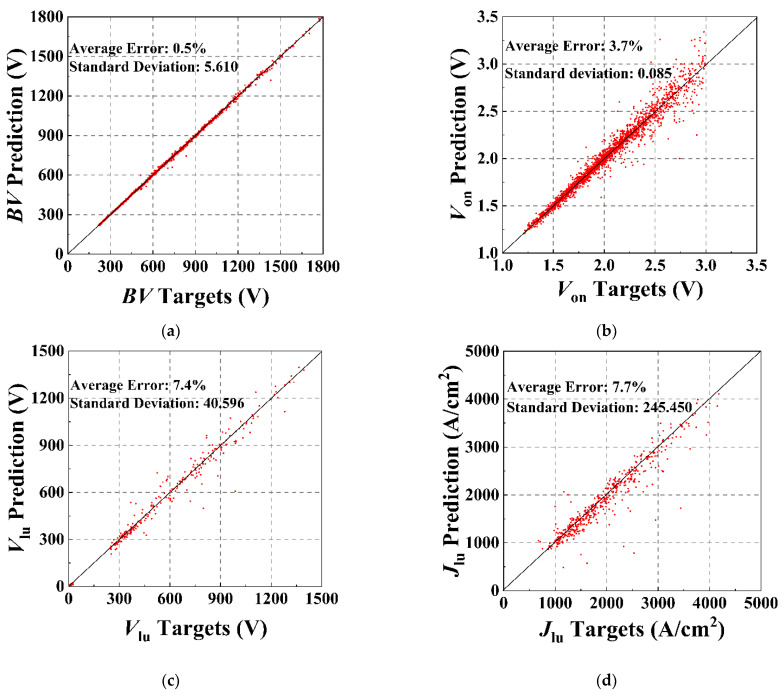

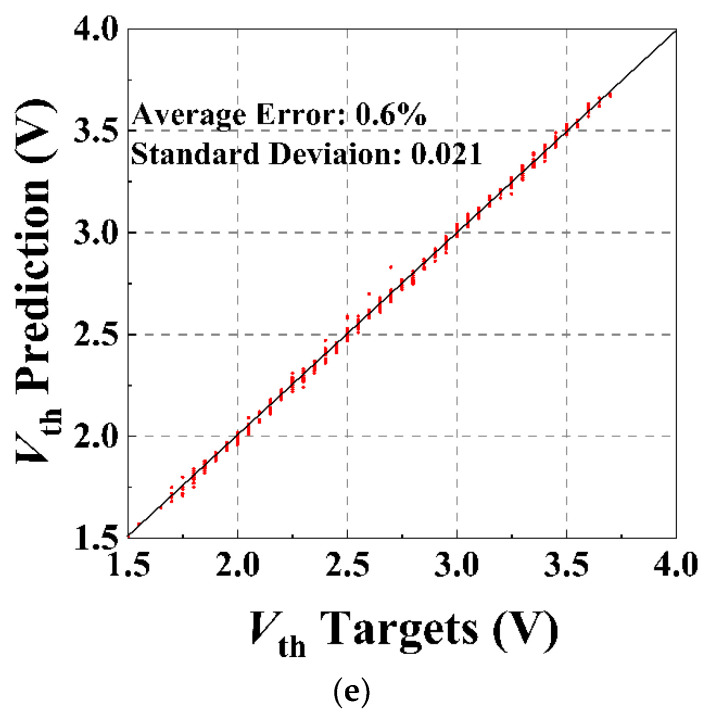
The scatter plots of the static characteristic prediction results for the testing sets of the IGBT: (**a**) *BV*, training sample size: 7600; (**b**) *V*_on_, training sample size: 7600; (**c**) *V*_lu_, training sample size: 4250; (**d**) *J*_lu_, training sample size: 4250; and (**e**) *V*_th_, training sample size: 1400.

Figure 4a–e are scatter plots showing the correlations between the target *BV*, *V*_on_, *V*_lu_, *J*_lu_, and *V*_th_ and those predicted by the ANN method, while the number of training samples used for the prediction of the different characteristic parameters is described in the caption text. The number of training samples is influenced by the convergence of the simulation and the complexity of the predicted characteristic parameters, which will be discussed more closely later in this paper. Compared to the TCAD simulation results, the average errors for *BV* prediction and *V*_th_ prediction can be controlled to within 1%. For IGBT, *BV* falls between the avalanche breakdown and the reach-through breakdown limits, and is governed by the open-base transistor breakdown phenomenon. The open-base transistor breakdown condition for the IGBT is given by Equation (2), where the α_pnp_ is the common-base current gain, which is related to the injection efficiency γ_E_, the base transport factor α_T_, and the multiplication coefficient *M*. By derivation, α_T_ and *M* are in turn related to *N*_d_ and *T*_d_ [21]. *V*_th_ is primarily associated with the doping concentration of the channel region, Nwell. The number of structural parameters affecting these two characteristic parameters is small, so the ANN can predict the *BV* and *V*_th_ of the IGBT very accurately. However, *V*_on_, *V*_lu_, and *J*_lu_ are closely related to on-resistance, and the parameters of the drift region, buffer region, and channel region all impact the on-resistance of the device. The more structural parameters affect the static characteristic parameter, the more complex the prediction of the characteristic parameter is. Nevertheless, the average errors of our proposed scheme are all within 8%, which proves that the number of layers and neurons of the framework is well set so that it has sufficient fitting ability and can avoid overfitting problems due to the high fitting ability.

(2) $$\alpha_{\mathrm{PNP}}={\left(\gamma_{E}\alpha_{T}\right)}_{\mathrm{PNP}}M=1$$

### 4.2. Comparison of Different Algorithms

To further emphasize the effectiveness of the ANN, we can compare the results of the ANN method with other predictors constructed using conventional regression algorithms, such as Gaussian process regression (GPR), support vector regression (SVR), and linear regression (LR). Table 2 shows the average errors of predictors constructed using different machine learning algorithms when predicting IGBT static characteristic parameters. The ANN achieves the most accurate predictions for each static characteristic parameter. In addition, it is worth noting that, except for the ANN, the average errors of the other three schemes for *V*_lu_ are large. This is because, for some structures with a very small *V*_lu_, these schemes fail to capture the nonlinear condition due to the high complexity of the data distribution, so it is difficult to give accurate prediction results. However, due to the setting of the ReLU function, the multi-layer ANN we trained has a strong nonlinear fitting ability, so the prediction result is more accurate compared to the other three schemes.

### 4.3. Effect of Sample Size on Results

**Figure 5:**
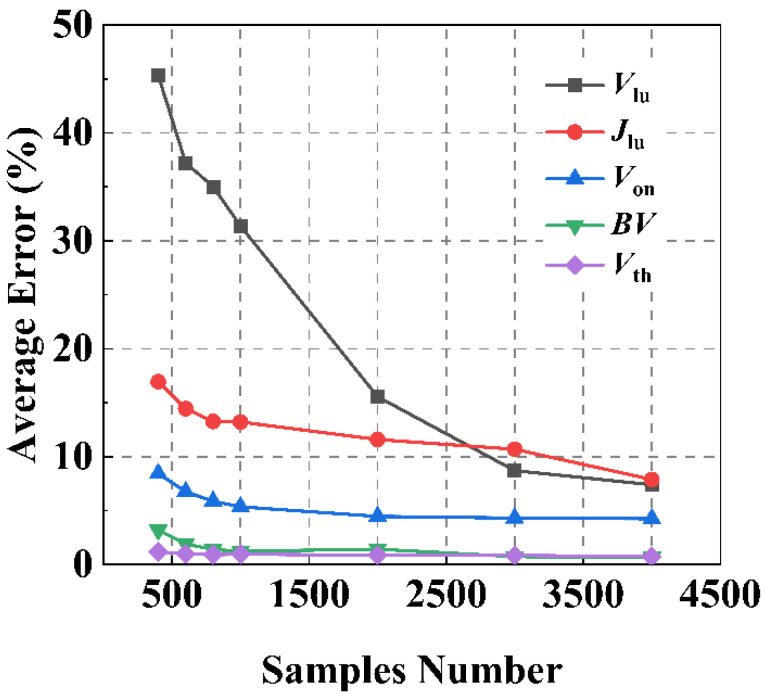
Curves of average error for static characteristic parameters varying with the number of training set samples based on the ANN method.

We can also analyze the influence of the number of training samples on the prediction results. The testing set consisted of 400 IGBT samples with different structural parameters, and the number of samples in the training set was increased from 400 to 4000. Figure 5 shows the variation curves of the ANN’s prediction average error for five static characteristic parameters as the number of samples in the training set increased.

It can be observed that the number of training set samples required to predict *J*_lu_ and *V*_lu_ are more than the other static characteristic parameters, and the average error is larger. This is because when the latch-up effect occurs, the internal parasitic NPN transistor of the IGBT turns on and the gate loses the ability to control the current. Under such conditions, the relationship between the device’s characteristics and the structural parameters is hard to capture, which assuredly increases the difficulty of the ANN’s prediction task.

### 4.4. Prediction Results with Changing Structural Parameters

**Figure 6:**
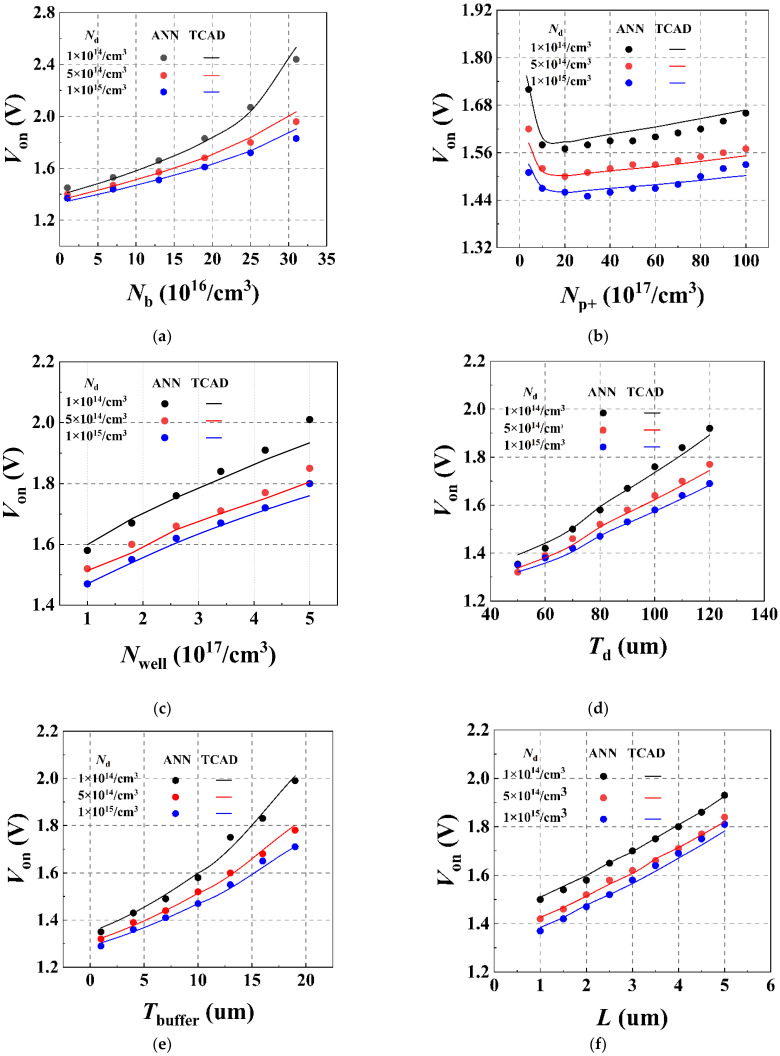
(**a**–**f**) Comparison of predicted results from ANN and TCAD for the *V*_on_ of devices under the condition that six structural parameters *N*_b_, *N*_p+_, *N*_well_, *T*_d_, *T*_buffer_, and *L* are changed, respectively.

Taking the *V*_on_ prediction as an example, we can further analyze the fitting results of the trained ANN relating to the relationship between the structural parameters and characteristic parameters of the IGBT. Figure 6a–f show the comparisons of the prediction results of the ANN method and the TCAD simulation with different structural parameters, including *N*_b_, *N*_p+_, *N*_well_, *T*_d_, *T*_buffer_, and *L*, respectively. Based on the initial structures, the structural parameters were sequentially changed. The initial structural parameters are shown in the caption. We have taken three different doping concentrations in the drift region into consideration.

As the figures show, compared with the TCAD simulation, the ANN method exhibits an accurate prediction ability in the presence of changes in the structural parameters, with the same trend as the TCAD simulation results. By comparing the points of three different colors in each figure, we can see that when other structural parameters remain unchanged, the larger *N*d is, the smaller *V*_on_ will be. This is because the on-state resistance of the drift region decreases with the increase of *N*_d_, and the anode voltage *V*_on_ required to reach a certain value of the anode current J_set_ decreases as well. In addition, larger values of the buffer region parameters *N*_b_ and *T*_buffer_ cause a decrease in the number of holes injected from the anode P+ region into the drift region, thereby weakening the conductance modulation effect, increasing the on-state resistance, and increasing *V*_on_. An increase in the structural parameters *N*_well_ and *L* in the channel region raises the threshold voltage, causing an increase in the on-state resistance and *V*_on_ of the device. As the figures show, the proposed scheme can fit the effects of various structural parameters on the static characteristic parameters of the device well.

### 4.5. Prediction Time and Efficiency

For the prediction using the ANN method, since the testing process is mathematically a matrix calculation between the input features and the updated parameters within the ANN, the prediction time of the ANN is within 0.1 s. Yet, under the same dataset, the simulation runtime for the TCAD tool is 328,653.6 s, which does not include the time spent manually setting up the device structure. The prediction speed is therefore increased by more than 10^7^ times compared with the TCAD simulation, and the convergence problem caused by unreasonable grid settings in the TCAD simulation is totally avoided, which is also beneficial for the efficiency when predicting the static characteristics parameters of the IGBTs with different structures.

Among the parameters of the initial structure, *N*^b^ is 1 × 10^16^ cm^−3^, *N*_well_ is 1 × 10^17^ cm^−3^, N_p+_ is 1 × 10^18^ cm^−3^, *T*_d_ is 80 µm, *T*_buffer_ is 10 µm, *L* is 2 µm, and three different cases with *N*_d_ of 1 × 10^14^ cm^−3^, 5 × 10^14^ cm^−3^, 1 × 10^15^ cm^−3^ are considered.

## 5. Conclusions

In this paper, we propose a multi-layer ANN predictive framework to predict multiple static characteristic parameters of an IGBT, such as *BV*, *V*_on_, *V*_lu_, *J*_lu_, and *V*_th_. Compared with the TCAD simulation tool, the trained ANN can achieve a speed increase of more than 10^7^ times while ensuring the average errors are less than 8% when predicting the static characteristic parameters. Since the prediction process is a numerical operation of the matrix, it avoids the convergence problem that often occurs with TCAD simulations. The scheme confirms that ANN can capture the effects of changes to a device’s structural parameters on a device’s characteristic parameters and help designers to predict the static characteristic parameters quickly and speed up the design process. The method is extensible and when the ANN is trained with more informative datasets, the ANN will have the ability to predict the characteristics of more complexly structured devices. In addition, the method is able to predict characteristic parameters that are closer to the experimental test values based on experimental data.

## Figures and Tables

**Table 1:** Structural parameters and ranges of the IGBT.

Structural Parameters	Range
N-drift doping, *N*_d_ (cm^−3^)	1 × 10^13^–1 × 10^15^
N-buffer doping, *N*_b_ (cm^−3^)	1 × 10^16^–5 × 10^17^
P-well doping, *N*_well_ (cm^−3^)	1 × 10^17^–5 × 10^17^
P^+^ anode doping, *N*_P+_ (cm^−3^)	1 × 10^17^–1 × 10^19^
N-drift thickness, *T*_d_ (µm)	50–120
N-buffer thickness, *T*_buffer_ (µm)	1–20
Channel length, *L* (µm)	1–5

**Table 2:** Average errors (%) of predictors constructed with different algorithms.

Characteristic Parameter	Machine Learning Algorithms			
	LR	SVR	GPR	ANN
*BV*	26.2	18.5	2.3	0.5
*V*_on_	56.1	41.7	12.0	3.7
*V*_th_	2.7	3.4	1.0	0.6
*V*_lu_	993.1	540.2	321.4	7.4
*J*_lu_	21.5	15.8	10.9	7.7

## Data Availability

The data presented in this study are available on request from the corresponding author.
